# Associations of triglyceride-glucose index with hyperuricemia among Royal Thai Army personnel

**DOI:** 10.1186/s12902-024-01542-3

**Published:** 2024-02-01

**Authors:** Sethapong Lertsakulbunlue, Tanatip Sangkool, Varathpavee Bhuriveth, Mathirut Mungthin, Ram Rangsin, Anupong Kantiwong, Boonsub Sakboonyarat

**Affiliations:** 1 Department of Pharmacology, Phramongkutklao College of Medicine, 10400 Bangkok, Thailand; 2 Department of Military and Community Medicine, Phramongkutklao College of Medicine, 10400 Bangkok, Thailand; 3 Medical cadet, Phramongkutklao College of Medicine, 10400 Bangkok, Thailand; 4 Department of Parasitology, Phramongkutklao College of Medicine, 10400 Bangkok, Thailand

**Keywords:** Triglyceride-glucose index, Obesity, Effect modification, Association, Relationship, Hyperuricemia

## Abstract

**Background:**

Hyperuricemia has placed an immense burden on the global healthcare system. Studies have discovered a close correlation between serum uric acid (SUA) and insulin resistance (IR). The objective of this investigation is to examine the association between the triglyceride-glucose (TyG) index, a simple surrogate for IR, and the presence of hyperuricemia.

**Methods:**

Between 2017 and 2021, an epidemiologic study was conducted on Royal Thai Army (RTA) personnel aged 35–60 years, involving a total of 231,286 participants. In the study, hyperuricemia was defined as a SUA level of 7 mg/dL and 6 mg/dL among male and female participants, respectively. Using linear regression analysis and logistic regression analysis, the association between the TyG index and SUA was determined.

**Results:**

A positive relationship was demonstrated between the TyG index and the SUA. Overall, SUA increased by 0.32 per unit of TyG index growth (95% CI: 0.31–0.32). In comparison with the first quartile, employees in the fourth TyG quartile had a greater likelihood of having hyperuricemia [adjusted odds ratio (AOR): 2.45, 95% CI: 2.38–2.52]. Effect modification by obesity on the association between the TyG index and SUA was observed (*P*-interaction < 0.001). Among individuals with obesity, compared with the first TyG index quartile, the AOR for hyperuricemia was 2.15 (95% CI: 2.06–2.25) and 2.14 (95% CI: 1.81–2.53) for the fourth quartile of the TyG index for males and females, respectively. However, for nonobese personnel, in comparison to the top quartile of the TyG index, the AOR for hyperuricemia was 2.73 (95% CI:2.61–2.84) and 5.03 (95% CI: 4.03–6.29) for the fourth quartile of the TyG index for males and females, respectively. Personnel in the fourth TyG index quartile revealed that the prevalence of hyperuricemia reached 44.2%.

**Conclusion:**

A robust positive association between the TyG index and SUA was illustrated among active-duty RTA personnel. Obesity was identified as a modifier influencing this relationship. Furthermore, individuals in the fourth quarter of the TyG index, regardless of their obesity status, could be considered appropriate candidates for screening SUA levels.

**Supplementary Information:**

The online version contains supplementary material available at 10.1186/s12902-024-01542-3.

## Background

A global increase in the prevalence of hyperuricemia and growing interest in the condition have been recognized [1, 2]. Uric acid is produced in the liver through the metabolism of purine compounds that are consumed by the body [3]. Although serum uric acid (SUA) and gout have a long-established relationship over a considerable period [4], recent research has also revealed links between SUA and other conditions such as metabolic syndromes, hypertension, nonalcoholic steatohepatitis disease, acute and chronic renal failure, type 2 diabetes, and insulin resistance (IR) [5–8]. Despite the potential ramifications of hyperuricemia, SUA testing is only recommended for specific groups of the population based on the Thai national health screening guidelines [9]. Consequently, the SUA testing process requires tools to determine which individuals should be subjected to the testing.

Hyperuricemia has been found to have a positive association with IR [5]. However, the current gold standard tests for IR, such as the invasive and expensive intravenous glucose tolerance test and euglycemic insulin clamp, pose limitations [10]. In 2010, as a quick and inexpensive substitute for measuring IR, the triglyceride-glucose (TyG) index was developed [10]. According to numerous studies, the TyG index and hyperuricemia are closely associated among diabetics, hypertensives, and general patients [11–14]. Nonetheless, it is worth noting that the existing studies on this topic have thus far only been carried out in China.

In a recent study, Lertsakulbunlue et al. highlighted the significance of the TyG index in anticipating higher aminotransferases in the Royal Thai Army (RTA) members, given the strong association of aminotransferases with IR [15]. Notably, the RTA population exhibited a considerably higher prevalence of hyperuricemia (31.4%) than other relevant studies conducted in Thailand [16, 17]. Furthermore, there is a dearth of information concerning the link between the TyG index and SUA levels, with the majority of investigations so far concentrating on the Chinese population. Building upon these findings, the study primary objective is to examine the associations between the TyG index and SUA levels, while also evaluating the predictive potentiality of the TyG index in identifying hyperuricemia among personnel within the RTA. This research makes a substantial contribution to the growing understanding of the TyG index’s potential use as a novel risk factor for hyperuricemia within the context of RTA workers.

## Methods

### Study design and subjects

**Fig. 1:**
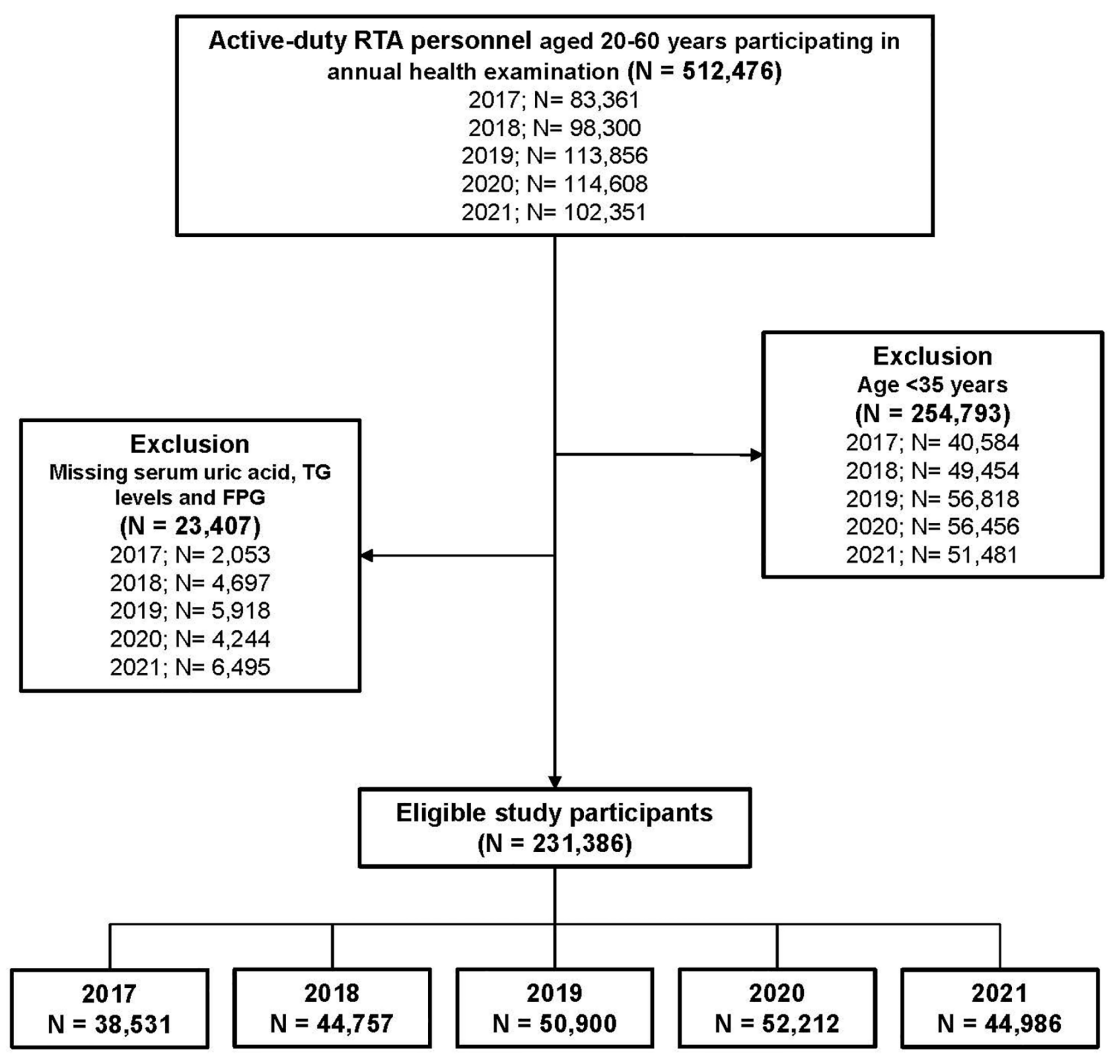
Flowchart of the enrolled participants

A serial cross-sectional study of 231,286 RTA personnel was conducted between 2017 and 2021. Sakboonyarat et al. reported the study’s specifics in another publication [18]. Before beginning the trial, approval was acquired by the Royal Thai Army Medical Department (RTAMED) in Thailand’s province of Bangkok. Following authorization from RTAMED, the database of yearly health examinations for RTA employees was extracted. The RTAMED is responsible for conducting yearly health exams for RTA employees across the country [18]. The RTAMED Centre in Bangkok received the findings of the medical exams. The study included active-service (nonretired) RTA employees aged 35–60. Since no serum uric acid test was done for participants under the age of 35, they were not enclosed in the study. Insufficient information from the laboratory tests for serum uric acid, fasting plasma glucose (FPG), and triglycerides (TG) also led to the exclusion of 23,407 patients from the analysis. The flow of enrolled participants is depicted in Fig. 1.

### Data collection

RTA personnel are subject to annual inspections by the Army Institute of Pathology, the Armed Forces Research Institute of Medical Sciences, and 37 RTA hospitals. To collect data on demographic and cognitive risk factors, a self-administered questionnaire was employed. The questionnaire comprised items addressing life span, gender, wellness allowance plans, daily workout, liquor consumption, and tobacco use. The smoking status was classified into three distinct groups, comprising (1) individuals who had never smoked, (2) ex-smokers who had abstained from smoking for 12 months, and (3) ongoing smokers. Likewise, alcohol consumption was categorized into three groups, namely (1), individuals who had never absorbed alcohol, (2) ex-drinkers who had refrained from alcohol consumption for 12 months, and (3) present consumers of alcohol. Physical activity for at least 30 min a day, three days a week, was deemed to be considered a daily workout. To determine the body mass index (BMI), anthropometric measurements encompassing height, weight, and systolic and diastolic blood pressure (SBP and DBP, respectively) were also performed.

Blood examinations were individually conducted and reported by certified RTA hospitals across Thailand, with trained technicians ensuring standardized and quality services accredited by the Healthcare Accreditation Institute. Hyperglycemia was classified as FPG ≤ 126 mg/dL for normal levels and FPG > 126 mg/dL for elevated levels. SBP ≥ 140 mmHg or DBP ≥ 90 mmHg was considered to be high blood pressure. Obesity was defined as a BMI of at least 25 kg/m^2^ [19, 20]. Males with elevated AST or ALT levels below 40 U/L and females with elevated AST or ALT levels below 35 U/L were considered to have high levels [15]. The TyG index was computed using the equation ln [TG (mg/dL)*FBG (mg/dL)/2] [10]. For men, hyperuricemia was deemed to be below 7 mg/dL, whereas, for women, it was below 6 mg/dL [1].

### Statistical analyses

A statistical examination was conducted by employing StataCorp, 2021, *Stata Statistical Software: Release 17*, College Station, TX: StataCorp LLC. To characterize the study subjects, population characteristic frequency distributions were created. A normal distribution was used to depict continuously distributed data, characterized by a mean and standard deviation (SD), whereas categorical variables were shown as percentages. For parameters exhibiting a nonnormal distribution, the median and interquartile range (IQR) were presented. The interchange between the mean SUA and baseline characteristics was assessed using appropriate statistical tests such as Student’s *t*-test and analysis of variance (ANOVA). The assumptions of the linear regression model were tested. We employed multivariable linear regression analysis to explore the association between TyG index and SUA, which was presented as adjusted β coefficient and 95% confidence interval (CI). Additionally, a multivariable logistic regression model was utilized to evaluate the association between TyG index and hyperuricemia, which was presented as adjusted odds ratio (AOR), with 95% CI.

Furthermore, the interaction between obesity (BMI ≥ 25 kg/m^2^) and the association of the TyG index with SUA were also examined. The statistical tests were performed with a two-sided approach and outcomes were considered statistically significant if the *P*-value was less than 0.05. The study identified the TyG threshold for predicting hyperuricemia by calculating Youden’s J statistic from the receiver operating characteristic (ROC) curve. Afterward, the area under the curve (AUC) of the ROC was calculated.

In the present study, data regarding participants’ medication usage was not gathered, limiting further insights into the consistent association between TyG and hyperuricemia. Although potential confounders were adjusted for in the final model, the possibility of residual confounding effects remains. Consequently, we conducted a sensitivity analysis to account for unmeasured confounding, utilizing E-values estimated by the evalue package [21, 22].

## Results

### Baseline characteristics of participants

The demographics, behaviors, and laboratory traits of 231,286 RTA personnel are displayed in Table 1. Most RTA personnel were male (89.4%) and their average age was 47.4 ± 7.5 years. More than half lived in the central region, and 97.9% were covered by the civil servant medical benefits program. Roughly, two-thirds of the participants currently consumed alcohol. The average BMI was 25.3 ± 3.7 kg/m^2^ and 47.6% were obese. The subjects’ respective mean SBP and DBP values were 131.3 ± 16.8 mmHg and 81.2 ± 11.7 mmHg. The median (IQR) FPG and TG levels were 95 (88 to 105) and 133 (92 to 197) mg/dL, respectively. Both the mean and median of the TyG index were 8.8. Finally, the average SUA level was 6.3 ± 1.5, with a prevalence of hyperuricemia at 31.4%.

**Table 1:** Demographic, behavioral characteristics and laboratory tests (*N* = 231,386)

Characteristics	Total
	*n* (%)
**No. of participants**	231,286 (100.0)
**Age (years)**	
35–39	47,641 (20.6)
40–44	43,538 (19.0)
45–49	37,481 (16.2)
50–54	46,746 (20.2)
55–60	55,980 (24.0)
Mean ± SD	47.4 ± 7.5
**Regions**	
Central	133,316 (57.6)
Northeast	40,116 (17.3)
North	35,378 (15.3)
South	22,576 (9.8)
**Health Scheme**	
Civil servant medical benefits	226,406 (97.9)
Social Security	3239 (1.4)
Universal Coverage	1741 (0.8)
**Current smoking**	
No	165,224 (73.0)
Yes	61,207 (27.0)
**Current alcohol drinking**	
No	81,203 (35.7)
Yes	146,458 (64.3)
**Regular exercise**	
No	96,414 (42.4)
Yes	130,785 (57.6)
**Body mass index (kg/m** ^**2**^ **)**	
Mean ± SD	25.3 ± 3.7
**Systolic blood pressure (mmHg)**	
Mean ± SD	131.3 ± 16.8
**Diastolic blood pressure (mmHg)**	
Mean ± SD	81.2 ± 11.7
**Fasting plasma glucose (mg/dL)**	
< 126	207,975 (89.9)
≥ 126	23,411 (10.1)
Median (IQR)	95 (88–105)
**Fasting triglyceride (mg/dL)**	
Median (IQR)	133 (92–197)
**Triglyceride-glucose index**	
Quartile 1 (< 8.37)	57,870 (25.0)
Quartile 2 (8.37–8.78)	57,823 (25.0)
Quartile 3 (8.79–9.23)	57,854 (25.0)
Quartile 4 (> 9.23)	57,839 (25.0)
Mean ± SD	8.8 ± 0.7
Median (IQR)	8.8 (8.4–9.2)
**Uric acid (mg/dL)**	
< 7 in male or < 6 in female	158,765 (68.6)
≥ 7 in male or ≥ 6 in female	72,621 (31.4)
Mean ± SD	6.3 ± 1.5
Median (IQR)	6.3 (5.3–7.3)

SD: standard deviation, IQR: interquartile range

### Relationship between baseline characteristics, TyG Index, and serum uric acid

The relationship between characteristics, TyG index, and SUA is presented in Table 2. Differences in the average SUA across TyG index quartiles were noticed (*P*-value < 0.001). In addition, the prevalence of hyperuricemia increased as the TyG index quartiles rose, and the prevalence (44.2%) was the highest in the fourth quartile. Hyperuricemia was more common in men (33.7%) than in women (12.4%). In addition, the prevalence of hyperuricemia in the obese group was 38.6%; conversely, the frequency of hyperuricemia was 24.8% in the nonobese group.

**Table 2:** Relationship between characteristics, laboratory tests and serum uric (*N* = 231,386)

Variables	Serum uric	*P*-value	Normouricemia	Hyperuricemia	*P*-value
	Mean ± SD		*n* (%)	*n* (%)	
**No. of participants**	6.3 ± 1.5		158,765 (68.6)	72,621 (31.4)	
**Sex**		< 0.001^a^			< 0.001^c^
Male	6.5 ± 1.5		137,167 (66.4)	69,569 (33.7)	
Female	4.8 ± 1.1		21,598 (87.6)	3052 (12.4)	
**Age (years)**		< 0.001^b^			< 0.001^c^
35–39	6.5 ± 1.6		30,951 (65.0)	16,690 (35.0)	
40–44	6.4 ± 1.6		29,152 (67.0)	14,386 (33.8)	
45–49	6.3 ± 1.5		26,096 (69.6)	11,385 (30.4)	
50–54	6.3 ± 1.5		32,749 (70.1)	13,997 (29.9)	
55–60	6.2 ± 1.5		39,817 (71.1)	16,163 (28.9)	
**Regions**		< 0.001^b^			< 0.001^c^
Central	6.3 ± 1.5		92,925 (69.7)	40,391 (30.3)	
Northeast	6.3 ± 1.6		27,772 (69.2)	12,344 (30.8)	
North	6.5 ± 1.6		23,217 (65.6)	12,161 (34.4)	
South	6.5 ± 1.5		14,851 (65.8)	7725 (34.2)	
**Health Scheme**		< 0.001^b^			< 0.001^c^
Civil servant medical benefits	6.4 ± 1.5		154,629 (68.3)	71,777 (31.7)	
Social Security	5.6 ± 1.5		2633 (81.3)	606 (19.7)	
Universal Coverage	5.2 ± 1.6		1503 (86.3)	238 (13.7)	
**Current smoking**		< 0.001^a^			< 0.001^c^
No	6.3 ± 1.5		114,325 (69.2)	50,899 (30.8)	
Yes	6.5 ± 1.5		41,240 (67.4)	19,967 (32.6)	
**Current alcohol drinking**		< 0.001^a^			< 0.001^c^
No	6.1 ± 1.6		59,279 (73.0)	21,924 (27.0)	
Yes	6.5 ± 1.5		97,047 (66.3)	49,411 (33.7)	
**Regular exercise**		0.031^a^			< 0.001^c^
No	6.3 ± 1.6		65,758 (68.2)	30,656 (31.8)	
Yes	6.3 ± 1.5		90,185 (69.0)	40,600 (31.0)	
**Body mass index (kg/m** ^**2**^ **)**		< 0.001^b^			< 0.001^c^
18.5–22.9	5.9 ± 1.5		47,228 (79.3)	12,321 (20.7)	
< 18.5	5.5 ± 1.6		2765 (83.6)	541 (16.4)	
23.0-24.9	6.3 ± 1.5		41,145 (70.6)	17,165 (29.4)	
25.0-29.9	6.6 ± 1.5		54,506 (62.6)	32,587 (37.4)	
≥ 30	6.8 ± 1.6		13,121 (56.7)	10,007 (43.3)	
**Systolic blood pressure (mmHg)**		< 0.001^a^			< 0.001^c^
< 140	6.2 ± 1.5		121,420 (71.0)	49,719 (29.1)	
≥ 140	6.6 ± 1.5		37,345 (62.0)	22,902 (38.0)	
**Diastolic blood pressure (mmHg)**		< 0.001^a^			< 0.001^c^
< 90	6.2 ± 1.5		128,238 (71.1)	52,146 (28.9)	
≥ 90	6.7 ± 1.6		30,527 (59.9)	20,475 (40.2)	
**Aspartate aminotransferase (U/L)**		< 0.001^a^			< 0.001^c^
< 40 in male or < 35 in female	6.3 ± 1.5		141,220 (70.2)	59,848 (29.8)	
≥ 40 in male or ≥ 35 in female	6.8 ± 1.7		17,545 (57.9)	12,773 (42.1)	
**Alanine aminotransferase (U/L)**		< 0.001^a^			< 0.001^c^
< 40 in male or < 35 in female	6.2 ± 1.5		124,021 (71.7)	48,849 (28.3)	
≥ 40 in male or ≥ 35 in female	6.7 ± 1.6		34,744 (59.4)	23,772 (40.6)	
**Fasting plasma glucose (mg/dL)**		< 0.001^a^			< 0.001^c^
< 126	6.4 ± 1.5		141,510 (68.0)	66,465 (32.0)	
≥ 126	6.1 ± 1.6		17,255 (73.7)	6156 (26.3)	
**Fasting triglyceride (mg/dL)**		< 0.001^a^			< 0.001^c^
< 150	6.0 ± 1.4		102,904 (76.3)	31,983 (23.7)	
≥ 150	6.8 ± 1.5		55,861 (57.9)	40,638 (42.1)	
**Triglyceride-glucose index**		< 0.001^b^			< 0.001^c^
Quartile 1 (< 8.37)	5.8 ± 1.4		47,333 (81.8)	10,537 (18.2)	
Quartile 2 (8.37–8.78)	6.2 ± 1.4		41,871 (72.4)	15,952 (27.6)	
Quartile 3 (8.79–9.23)	6.5 ± 1.5		37,261 (64.4)	20,593 (35.6)	
Quartile 4 (> 9.23)	6.8 ± 1.6		32,300 (55.8)	25,539 (44.2)	

^a^student t-test, ^b^ANOVA, ^c^chi-square

**Table 3:** Overall and Obese-specific multivariable linear regression analysis of serum uric acid and triglyceride-glucose (TyG) index stratified by sex

**Variables**	**Serum uric acid**								
	**Overall**			**Male**			**Female**		
	**Adjusted β Coefficient**	**95% CI**	***P*** **-value**	**Adjusted β Coefficient**	**95% CI**	***P*** **-value**	**Adjusted β Coefficient**	**95% CI**	***P*** **-value**
**Overall** ^**a**^									
**TyG index**	0.32	0.31–0.32	< 0.001	0.31	0.30–0.32	< 0.001	0.33	0.30–0.35	< 0.001
**TyG index (Quartiles)**									
Quartile 1 (< 8.37)	ref			ref			ref		
Quartile 2 (8.37–8.78)	0.24	0.23–0.26	< 0.001	0.23	0.21–0.25	< 0.001	0.23	0.20–0.27	< 0.001
Quartile 3 (8.79–9.23)	0.44	0.42–0.46	< 0.001	0.43	0.41–0.44	< 0.001	0.45	0.41–0.49	< 0.001
Quartile 4 (> 9.23)	0.61	0.59–0.63	< 0.001	0.61	0.59–0.63	< 0.001	0.46	0.41–0.51	< 0.001
**Obese** ^**b**^ *									
**TyG index**	0.26	0.25–0.27	< 0.001	0.25	0.24–0.27	< 0.001	0.31	0.26–0.35	< 0.001
**TyG index (Quartiles)**									
Quartile 1 (< 8.37)	ref			ref			ref		
Quartile 2 (8.37–8.78)	0.24	0.21–0.27	< 0.001	0.23	0.20–0.26	< 0.001	0.23	0.17–0.30	< 0.001
Quartile 3 (8.79–9.23)	0.41	0.39–0.44	< 0.001	0.40	0.37–0.43	< 0.001	0.45	0.38–0.51	< 0.001
Quartile 4 (> 9.23)	0.56	0.53–0.59	< 0.001	0.56	0.53–0.58	< 0.001	0.43	0.35–0.51	< 0.001
**Non-obese** ^**b**^ *									
**TyG index**	0.39	0.37–0.40	< 0.001	0.38	0.36–0.39	< 0.001	0.41	0.38–0.44	< 0.001
**TyG index (Quartiles)**									
Quartile 1 (< 8.37)	ref			ref			ref		
Quartile 2 (8.37–8.78)	0.24	0.22–0.26	< 0.001	0.23	0.21–0.25	< 0.001	0.26	0.22–0.30	< 0.001
Quartile 3 (8.79–9.23)	0.47	0.45–0.49	< 0.001	0.46	0.43–0.48	< 0.001	0.54	0.48–0.59	< 0.001
Quartile 4 (> 9.23)	0.69	0.67–0.72	< 0.001	0.69	0.66–0.71	< 0.001	0.63	0.55–0.70	< 0.001

^a^Adjusted for age, sex, body mass index, region, scheme, year, smoking status, alcohol drinking, exercise, systolic blood pressure, diastolic blood pressure, aspartate aminotransferase, alanine aminotransferase

^b^Adjusted for age, sex, region, scheme, year, smoking status, alcohol drinking, exercise, systolic blood pressure, diastolic blood pressure, aspartate aminotransferase, alanine aminotransferase

*P for interaction (obesity and TyG index) < 0.05

### Linear regression analysis for the associations of triglyceride-glucose index with hyperuricemia

**Fig. 2:**
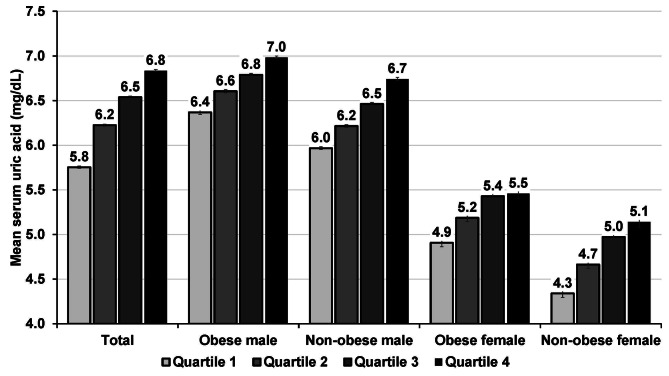
Mean serum uric acid stratified by triglyceride-glucose (TyG) index quartiles and by obese and sex

Figure 2 demonstrates the obese- and sex-specific mean SUA by TyG index quartiles. For the first to fourth TyG index quartiles, the mean SUA values were 5.8, 6.2, 6.5, and 6.8 mg/dL, respectively. Males had a high SUA of 7.0 mg/dL and 6.7 mg/dL, respectively, in both obese and nonobese groups. Table 3 delineates the multivariable linear regression analysis of SUA and TyG index stratified by sex. The TyG index and SUA have a dose-response relationship (adjusted β = 0.32, 95% CI: 0.31–0.32) when potential factors such as age, gender, BMI, district, scheme, year enrolled, smoking status, alcohol intake, physical activity, SBP, DBP, AST, and ALT have been taken into account. In addition, in comparison with the first quartile, in the fourth quartile, the adjusted β coefficients for SUA were 0.61 (95% CI: 0.59–0.63) and 0.46 (95% CI: 0.41 to 0.51) for males and females, respectively.

Obesity was spotted to have an effect modification impact on the relationship between the TyG index and SUA (*P*-interaction < 0.001). The adjusted β coefficients for SUA reached 0.25 (95% CI: 0.24 to 0.27) and 0.31 (95% CI: 0.26 to 0.35) for male and female participants who were obese, respectively. Conversely, a stronger interaction between the TyG index and SUA was noted among individuals without obese status. Among nonobese participants, the adjusted β coefficients for SUA were 0.38 (95% CI: 0.36 to 0.39) and 0.41 (95% CI: 0.38 to 0.44) for both males and females, respectively.

**Fig. 3:**
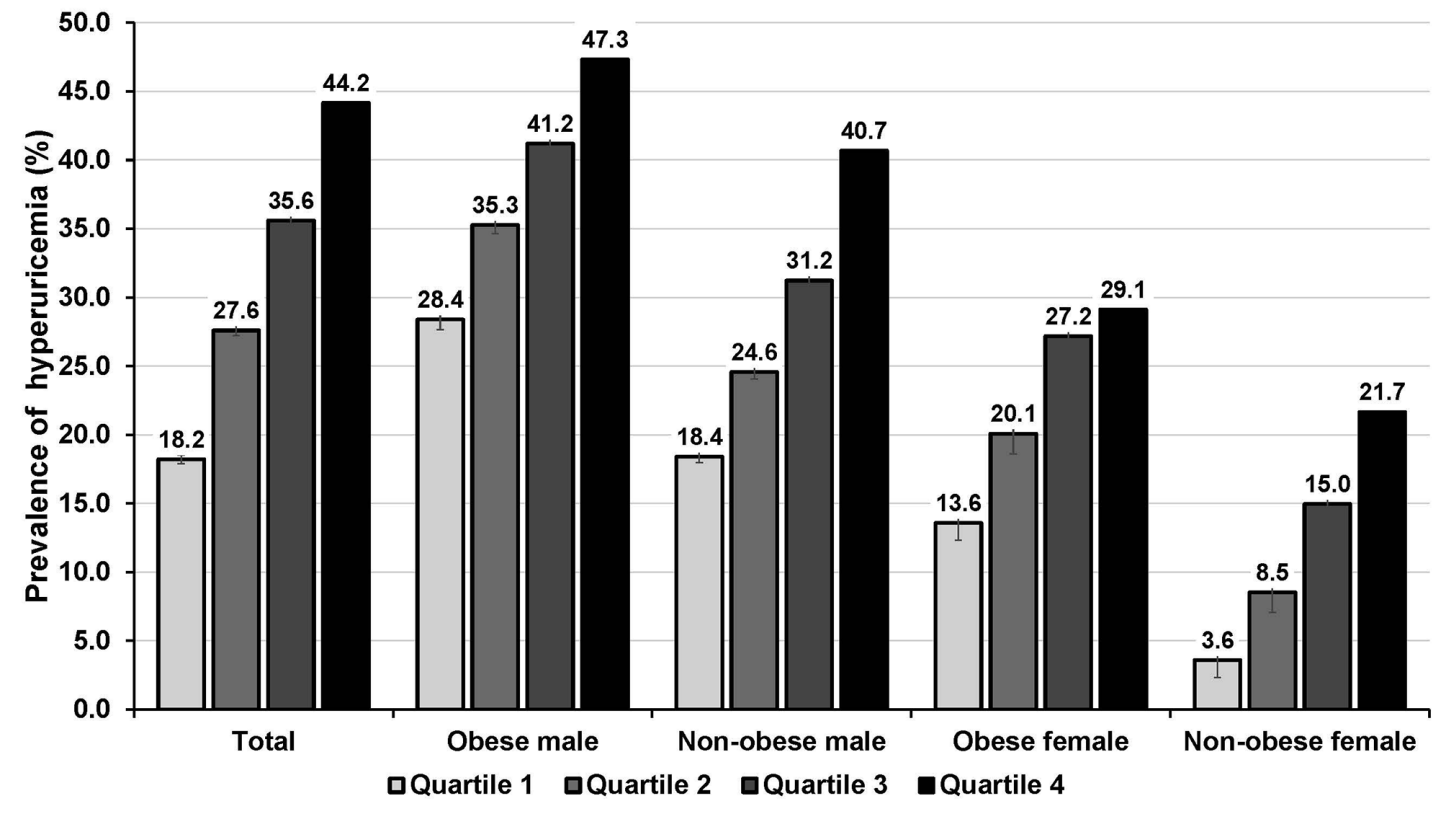
Prevalence of hyperuricemia stratified by triglyceride-glucose (TyG) index quartiles and by obese and sex

Figure 3 illustrates the obese- and sex-specific prevalence of hyperuricemia categorized by TyG index quartiles. Generally, the predominance of hyperuricemia amounted to 44.2% in the fourth quartile and 18.2% in the first quartile. Males in the fourth TyG index quartiles with and without obese status reflected a substantial prevalence of hyperuricemia (47.3 and 40.7%, respectively).

### ROC curve analysis of the TyG Index to Anticipate Hyperuricemia

**Fig. 4:**
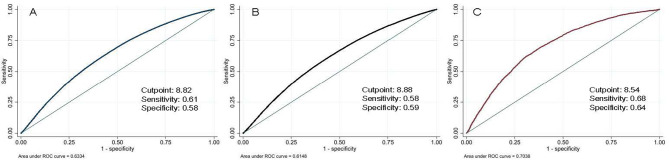
ROC curve analysis of the TyG index prediction of hyperuricemia: **(A)** overall, **(B)** male and **(C)** female

The TyG index’s ability to anticipate hyperuricemia is exhibited in Fig. 4. The ideal cutoff point for predicting hyperuricemia using the TyG index is 8.82, as shown in Fig. 4a (sensitivity 0.61, specificity 0.58). The total AUC for this prediction is 0.63. Male and female ROC curves are represented in Fig. 4b and c, respectively. Using an optimal cutoff value of 8.54, the AUC was 0.70 (sensitivity 0.68, specificity 0.64) when predicting hyperuricemia among females with the TyG index. The optimum TyG index cutoff point for predicting hyperuricemia among males was 8.88 (sensitivity 0.58, specificity 0.59). In Supplementary Table 1, a multivariable logistic regression analysis employing the TyG index’s ideal cutoff value association to hyperuricemia is presented.

### Logistic regression analysis for the associations of triglyceride-glucose index with Hyperuricemia

**Fig. 5:**
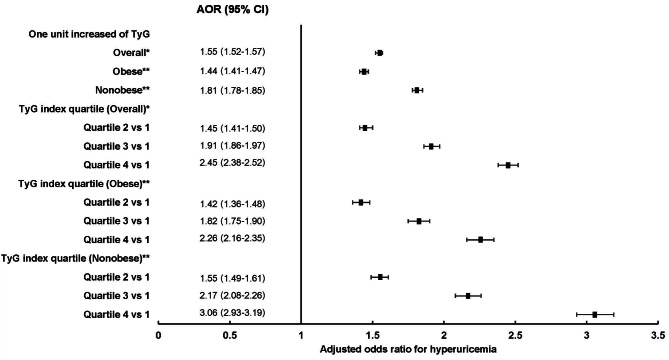
Forest plot of the obese-specific multivariable regression analysis of the association of the Triglyceride-glucose (TyG) index with hyperuricemia. *Adjusted for age, sex, body mass index, region, scheme, year, smoking status, alcohol drinking, exercise, systolic blood pressure, diastolic blood pressure, aspartate aminotransferase, alanine aminotransferase. **Adjusted for the same as * but excluding body mass index

Figure 5 displays the forest plot of multivariable logistic regression analysis of the link of the TyG index with hyperuricemia for obese individuals. After accounting for age, sex, BMI, region, scheme, year of enrollment, smoking status, drinking habits, exercise routine, SBP, DBP, AST, and ALT, an increase of one unit in the TyG index induced a 1.55 AOR for hyperuricemia (95% CI: 1.52 to 1.57). TyG index quartiles and hyperuricemia were found to be correlated in terms of dose. As indicated in Supplementary Table 2, RTA employees in the fourth quartile had substantially greater probabilities of having hyperuricemia than those in the first quartile (AOR: 2.39, 95% CI: 2.31 to 2.46 for men; AOR: 2.86, 95% CI: 2.49 to 3.27 for women; *P* < 0.001 for both).

Effect modification by obesity on the association of the TyG index with hyperuricemia was also recognized, particularly in women. For participants with obese status, the AOR was 1.44 (95% CI: 1.41 to 1.47), while for participants without obese status AOR was 1.81 (95% CI: 1.78 to 1.85). A stronger association between TyG index quartiles and hyperuricemia was highlighted among the nonobese group, particularly females. In the obese population, in comparison with the first TyG index quartile, the AOR for hyperuricemia was 2.15 (95% CI: 2.06 to 2.25) and 2.14 (95% CI: 1.81 to 2.53) in the fourth quartile of the TyG index for men and women, respectively. On the other hand, for personnel with nonobese status, in comparison with the first TyG index quartile, the AOR for hyperuricemia was 2.73 (95% CI: 2.61 to 2.84) and 5.03 (95% CI: 4.03 to 6.29) in the fourth quartile of the TyG index for both men and women, independently.

## Discussion

The current study involved an extensive cross-sectional investigation carried out in RTA staff. It successfully established a dose-response correlation between the TyG index and hyperuricemia. Additionally, it explained how obesity has an altered impact on the interaction between the TyG index and hyperuricemia, particularly among females. Furthermore, the study demonstrated a dose-response connection between the profusion of the TyG index quartiles and hyperuricemia. Moreover, when employed to predict hyperuricemia, the TyG index displayed fair predictive capability. As far as we are aware, this study is the first and most comprehensive analysis of the relationship between the TyG index and hyperuricemia in a Thai population.

This epidemiological study unveiled a pervasiveness rate of hyperuricemia at 31.4%, which is comparatively higher than that marked in the normal Thai population [16, 17]. This occurrence can be attributed to the relatively elevated prevalence of metabolic syndrome in RTA staff in contrast to the normal Thai population [23]. Additionally, a notable predominance of males was spotted within the RTA personnel group, and it is known that males tend to exhibit a greater pervasiveness of psychological risk parameters, encompassing smoking and drinking consumption [24, 25].

IR and metabolic syndrome are widely recognized for their closely intertwined pathophysiological connection with hyperuricemia [26]. IR triggers the conversion of intermediates from glycolysis and the pentose phosphate pathways into ribose pyrophosphate and 5-ribose phosphate, thereby promoting the production of SUA [27]. Furthermore, IR can exacerbate inflammatory cascades in the liver, potentially leading to liver dysfunction and increased SUA production [28]. Additionally, a buildup of uric acid in the bloodstream can result from IR-related hyperinsulinemia impairing renal urate excretion [13, 29]. The TyG index, a simple and affordable substitute for IR, uncovered a dose-response relationship with SUA in the current study.

In line with the existing study, recent research conducted in general Chinese populations has also discussed a positive connection between the TyG index and hyperuricemia [14, 30, 31]. After adjusting possible confounding factors, a linear dose-response relationship was pinpointed, with a 0.24 mg/dL increase in SUA for each 1-unit rise in the TyG index [31]. Additionally, an investigation in China manifested that the TyG index exhibited a stronger correlation with hyperuricemia than obesity indices [14]. Furthermore, Gu et al. executed a reflective longitudinal analysis of a Chinese population that initially did not present hyperuricemia during baseline. They found that a greater quartile of the TyG index was related to an elevated incidence of hyperuricemia among both males and females [30].

In this research, a significantly higher pervasiveness of hyperuricemia was scrutinized between obese and nonobese individuals. Additionally, obesity was detected to alter the TyG index’s connection to hyperuricemia. These results are in agreement with related studies that used information from the National Health and Nutrition Examination Survey (NHANES) [32]. The TyG index and hyperuricemia were found to be significantly correlated, and the authors also perceived that obesity mediated the link between the TyG index and SUA levels [32]. In contrast to persons with obesity, a retrospective cohort research remarked a substantial connection between the TyG index and the occurrence of type 2 diabetes in the nonobese population [33].

Conversely, a study conducted in the general population of China documented a consistent association when stratified by BMI [31]. This discrepancy in outcomes could be explained by the study’s smaller sample size and lower mean BMI (24.56 kg/m^2^) [31]. Moreover, the SUA level in their study was comparatively lower at 4.32 mg/dL, as opposed to 6.30 mg/dL in this study. Additionally, variations in race and diet might have also contributed to this discrepancy. In the present investigation, obese workers in the fourth quartile of the TyG index exposed the maximal predominance of hyperuricemia. Specifically, 47.3% of males and 29.1% of females with obese status in the fourth TyG index quartile exhibited hyperuricemia. Additionally, a weaker interrelation was declared between the TyG index and hyperuricemia among participants with obese status.

Obesity has been widely recognized as having a direct positive impact on hyperuricemia [4, 7]. A recent genetic probe has affirmed that increased body fat was associated with higher SUA levels [34]. Conversely, there is a perspective that suggests elevated SUA levels may contribute to increased fat storage [35]. Furthermore, genome-wide association studies have identified several gene variants related to urate transporters, interacting proteins, and enzymes involved in metabolic pathways, which play a role in the development of hyperuricemia and its link to obesity [36]. Consequently, a bidirectional relationship between obesity and hyperuricemia may exist. As a result, the effect of obesity could obscure the impact of a high TyG index, thereby diluting its association with hyperuricemia among obese participants.

Adipose tissues are known to release proinflammatory cytokines that have been shown to exacerbate IR [37, 38]. The inflated levels of IR observed among participants with obesity could potentially further weaken the connection between a sublime TyG index and SUA, especially in women who typically have a higher percentage of adipose tissue compared to men. This is especially relevant as BMI serves as a strong indicator linked to the primary marker of adipose tissue IR [39].

Another hypothesis suggests that variations may exist in the adipogenic capacities of preadipocytes among individuals [33]. Specifically, preadipocytes in the visceral fat depot have been distinguished to exhibit lower adipogenic capacity than those in the subcutaneous fat depot [40]. It is worth noting that visceral fat depot, especially in the liver, is crucial when discussing insulin resistance [41]. As a result, individuals with visceral fat obesity and fatty liver may not necessarily validate an excess in terms of BMI. Nevertheless, regular physical activity and long-term weight loss are still recommended for individuals with obesity, as research has confirmed that successful weight loss maintainers proved sustained improvements in insulin sensitivity [42].

In a recent study by Lertsakulbunlue et al., it was stated that a TyG index threshold of 8.89 for males and 8.57 for females could effectively predict elevated aminotransferase levels [15]. Accordingly, the present study unveiled similar TyG index edges of 8.88 for males and 8.54 for females as optimal cutoff points for identifying hyperuricemia. These findings assume that these specific cutoff points may be utilized to predict both elevated aminotransferase levels and hyperuricemia in RTA populations. A study implemented in China also reported a similar outcome in predicting hyperuricemia, with cutoff points of 8.58 (sensitivity 63.1%, specificity 74.3%) observed in the general population [14]. Comparing the TyG index to the Homeostatic Model Assessment for Insulin Resistance (HOMA-IR), another alternate measure for insulin resistance, individuals with nonobese status and type 2 diabetes mellitus proved TyG index’s superior predictive ability for hyperuricemia [11].

Although the optimal cutoff points for identifying hyperuricemia were determined, the sensitivity and specificity were relatively low, suggesting they might not be ready for implementation. However, a high TyG index is clearly associated with hyperuricemia. Prior studies have sought to enhance the TyG index’s sensitivity and specificity in predicting diabetes and insulin resistance by incorporating BMI, waist-to-height ratio, and waist circumference, yielding improved outcomes [43, 44]. Similarly, incorporating BMI into the model provides better predictive ability in this study (Supplementary Fig. 1). These variables have also been previously incorporated into models predicting hyperuricemia [45]. Moreover, these variables had clinical applicability and are routinely measured in clinical settings. Therefore, integrating the TyG index with other variables may benefit from a more accurate prediction of hyperuricemia.

Currently, there is no established pharmaceutical therapy to lower blood uric acid levels in asymptomatic hyperuricemia among the general population [46]. Additionally, in Thailand, the screening of SUA in the general population is not a common practice [9]. As a result, even though there may be connections with nonalcoholic steatohepatitis, acute and chronic renal failure, and cardiovascular diseases, the diagnosis and lifestyle modification of asymptomatic hyperuricemia can be subject to delays and potentially go unrecognized. Furthermore, subgroup analysis found a tremendous profusion of hyperuricemia among individuals in the fourth quartile of the TyG index, regardless of their obesity status (47% for the obese and 40% for the nonobese persons). Therefore, screening for SUA should be conducted in populations with elevated TyG index levels specifically those within the fourth TyG index quartile. This would enable prompt implementation of changes like adopting a urate-lowering diet, gradual slim-down, regular exercise, and alcohol curtailment [47]. Moreover, it is recommended that further studies be conducted on combining the TyG index with other factors to enhance its predictive capability for hyperuricemia.

### Strengths and limitations

Numerous notable advantages characterize this investigation. First of all, this is Thailand’s first in-depth study of the connection between the TyG index and SUA levels. Furthermore, the study involved a nationally representative sample of RTA workers, thereby enhancing the applicability of the data within the RTA population. The study also offers important information about the connection between TyG index quartiles and SUA levels, including the pervasiveness of hyperuricemia in RTA members. Additionally, obesity is identified as a modifier influencing the interchange between the TyG index and SUA levels. These findings have the potential to inform upcoming plans to prevent hyperuricemia.

The present study is subject to several limitations. Firstly, since the study is cross-sectional, a causal connection cannot be established between the TyG index and SUA levels. Secondly, the study sample primarily comprised active-duty RTA personnel, with a vast majority of male participants (approximately 90%). While this demographic reflects the composition of the RTA members, it is essential to acknowledge that this may potentially restrict the study’s external validity to other populations. Furthermore, as an observational study relying on data collected from health examination databases, the study has inherent limitations. The collection of behavioral risk variables might have lacked precision, and there was limited information on potential confounding factors, including participants’ underlying diseases and medication usage. Furthermore, data on uric acidlowering agents, which could be potential confounders affecting the association strength between the TyG index and hyperuricemia, was unavailable. Nevertheless, the sensitivity analysis results revealed a relatively high E-value, indicating a robust association between TyG and hyperuricemia, as shown in Supplementary Table 3.Furthermore, given our extensive data and the fact that many previous studies investigating hyperuricemia have not reported the use of uric acidlowering drugs, we believe that our results remain acceptable [31, 48–50]. In addition, the current study results are comparable to findings from other studies [3, 14, 31, 50]. Finally, due to the absence of serum insulin levels, HOMA-IR, which is another insulin resistance surrogate, could not be compared.

## Conclusion

The current study has uncovered the prevalence of hyperuricemia as a serious health concern within the population of RTA personnel, particularly among males and individuals who fall into the fourth quartile of the TyG index. It has demonstrated an effective favorable association between the TyG index and SUA levels, with obesity acting as an effect modifier. This finding highlights the potential of the TyG index as a possible novel risk factor for identifying hyperuricemia in RTA staff and suggests its potential usefulness in hyperuricemia screening efforts.

### Electronic supplementary material

Below is the link to the electronic supplementary material.

Supplementary Material 1

## Data Availability

The dataset is owned by the Royal Thai Army Medical Department and cannot be made public since it contains identifiable information. As a result, the dataset is subject to ethical constraints. Researchers who fulfill the requirements can access secret data from the Royal Thai Army Medical Department in Bangkok, Thailand.

## Abbreviations

SUA: Serum uric acid
IR: Insulin resistance
FPG: Fasting plasma glucose
TG: Triglyceride
TyG index: Triglyceride glucose index
AST: Aspartate aminotransferase
ALT: Alanine aminotransferase
BMI: Body mass index
SBP: Systolic blood pressure
DBP: Diastolic blood pressure
HT: Hypertension
AOR: Adjusted odds ratio
CI: Confidence interval
IQR: Interquartile range
SD: Standard deviation
