# Supplementary material for: Associations of triglyceride-glucose index with hyperuricemia among Royal Thai Army personnel
Source: BMC Endocr Disord. 2024 Feb 1;24:17. doi: 10.1186/s12902-024-01542-3 (PMC10832246; doi:10.1186/s12902-024-01542-3)
Supplement: Supplementary file 1 — Supplementary Material 1 [file 12902_2024_1542_MOESM1_ESM.docx]

**Supplementary File**

**Associations of Triglyceride-Glucose Index with Hyperuricemia among Royal Thai Army Personnel**

| Supplementary table 1. Overall and Obese-specific multivariable logistic regression analysis of serum uric acid and triglyceride-glucose (TyG) index stratified by sex using the TyG index optimal cutoff value to predict hyperuricemia. | Page 2 |
| --- | --- |
| Supplementary table 2. Overall and Obese-specific multivariable logistic regression analysis of serum uric acid and triglyceride-glucose (TyG) index stratified by sex. | Page 3 |
| Supplementary figure 1. Comparison of receiver operating characteristic curve between triglyceride-glucose (TyG) index only versus TyG with body mass index model. | Page 4 |
| Supplementary table 3. Sensitivity analysis for unmeasured confounding using E-value for odds ratio | Page 5 |
| Example of E-value interpretation | Page 6 |

| Supplementary table 1. Overall and Obese-specific multivariable logistic regression analysis of serum uric acid and triglyceride-glucose (TyG) index stratified by sex using the TyG index optimal cutoff value to predict hyperuricemia | | | | | | | | | |  |
| --- | --- | --- | --- | --- | --- | --- | --- | --- | --- | --- |
|  |  |  |  |  |  |  |  |  |  |  |
| **Variables** | **Serum uric acid** | | | | | | | | |  |
|  | **Overall** | | | **Male** | | | **Female** | | |  |
|  | **Adjusted odds ratio** | **95% CI** | ***p*-value** | **Adjusted odds ratio** | **95% CI** | ***p*-value** | **Adjusted odds ratio** | **95% CI** | ***p*-value** |  |
| **Overall^a^** | | | | | | | | | |  |
| **Low TyG index** | ref |  |  | ref |  |  | ref |  |  |  |
| **High TyG index** | 1.73 | 1.70-1.77 | <0.001 | 1.71 | 1.68-1.75 | <0.001 | 2.00 | 1.84-2.18 | <0.001 |  |
| **Obese^b^** | | | | | | | | | |  |
| **Low TyG index** | ref |  |  | ref |  |  | ref |  |  |  |
| **High TyG index** | 1.59 | 1.55-1.64 | <0.001 | 1.57 | 1.53-1.61 | <0.001 | 1.88 | 1.68-2.11 | <0.001 |  |
| **Non-obese^b^** | | | | | | | | | |  |
| **Low TyG index** | ref |  |  | ref |  |  | ref |  |  |  |
| **High TyG index** | 1.94 | 1.89-2.00 | <0.001 | 1.91 | 1.86-1.97 | <0.001 | 2.83 | 2.47-3.23 | <0.001 |  |
| ^a^Adjusted for age, sex, body mass index, region, scheme, year, smoking status, alcohol drinking, exercise, systolic blood pressure, diastolic blood pressure, aspartate aminotransferase, alanine aminotransferase | | | | | | | | | |  |
| ^b^Adjusted for age, sex, region, scheme, year, smoking status, alcohol drinking, exercise, systolic blood pressure, diastolic blood pressure, aspartate aminotransferase, alanine aminotransferase | | | | | | | | | |  |
| **^c^**Low TyG index is ≤8.82 and high TyG index is >8.82 for Overall, Low TyG index is ≤8.88 and high TyG index is >8.88 for males, Low TyG index is ≤8.54 and high TyG index is >8.54 for females | | | | | | | | | |  |

| Supplementary table 2. Overall and Obese-specific multivariable logistic regression analysis of serum uric acid and triglyceride-glucose (TyG) index stratified by sex | | | | | | |  |
| --- | --- | --- | --- | --- | --- | --- | --- |
|  |  |  |  |  |  |  |  |
| **Variables** |  | | | | | |  |
|  | **Male** | | | **Female** | | |  |
|  | **Adjusted odds ratio** | **95% CI** | ***P*-value** | **Adjusted odds ratio** | **95% CI** | ***P*-value** |  |
| **Overall^a^** | | | | | | |  |
| **TyG index** | 1.53 | 1.50-1.55 | <0.001 | 1.94 | 1.80-2.09 | <0.001 |  |
| **TyG index (Quartiles)** |  |  |  |  |  |  |  |
| Quartile 1 (<8.37) | ref |  |  | ref |  |  |  |
| Quartile 2 (8.37-8.78) | 1.41 | 1.37-1.45 | <0.001 | 1.79 | 1.60-2.00 | <0.001 |  |
| Quartile 3 (8.79-9.23) | 1.84 | 1.78-1.89 | <0.001 | 2.65 | 2.36-2.97 | <0.001 |  |
| Quartile 4 (>9.23) | 2.39 | 2.31-2.46 | <0.001 | 2.86 | 2.49-3.27 | <0.001 |  |
| **Obese^b^*** | | | | | | |  |
| **TyG index** | 1.40 | 1.37-1.43 | <0.001 | 1.62 | 1.48-1.78 | <0.001 |  |
| **TyG index (Quartiles)** |  |  |  |  |  |  |  |
| Quartile 1 (<8.37) | ref |  |  | ref |  |  |  |
| Quartile 2 (8.37-8.78) | 1.38 | 1.31-1.44 | <0.001 | 1.46 | 1.26-1.69 | <0.001 |  |
| Quartile 3 (8.79-9.23) | 1.74 | 1.66-1.82 | <0.001 | 2.08 | 1.79-2.41 | <0.001 |  |
| Quartile 4 (>9.23) | 2.15 | 2.06-2.25 | <0.001 | 2.14 | 1.81-2.53 | <0.001 |  |
| **Non-obese^b^*** | | | | | | |  |
| **TyG index** | 1.71 | 1.67-1.74 | <0.001 | 2.73 | 2.43-3.06 | <0.001 |  |
| **TyG index (Quartiles)** |  |  |  |  |  |  |  |
| Quartile 1 (<8.37) | ref |  |  | ref |  |  |  |
| Quartile 2 (8.37-8.78) | 1.41 | 1.35-1.47 | <0.001 | 2.13 | 1.81-2.52 | <0.001 |  |
| Quartile 3 (8.79-9.23) | 1.92 | 1.85-2.00 | <0.001 | 3.77 | 3.16-4.51 | <0.001 |  |
| Quartile 4 (>9.23) | 2.73 | 2.61-2.84 | <0.001 | 5.03 | 4.03-6.29 | <0.001 |  |
| ^a^Adjusted for age, sex, body mass index, region, scheme, year, smoking status, alcohol drinking, exercise, systolic blood pressure, diastolic blood pressure, aspartate aminotransferase, alanine aminotransferase | | | | | | |  |
| ^b^Adjusted for age, sex, region, scheme, year, smoking status, alcohol drinking, exercise, systolic blood pressure, diastolic blood pressure, aspartate aminotransferase, alanine aminotransferase | | | | | | |  |
| **P* for interaction (obesity and TyG index) <0.05 | | | | | | |  |

Supplementary figure 1. Comparison of receiver operating characteristic curve between triglyceride-glucose (TyG) index only versus TyG with body mass index model.


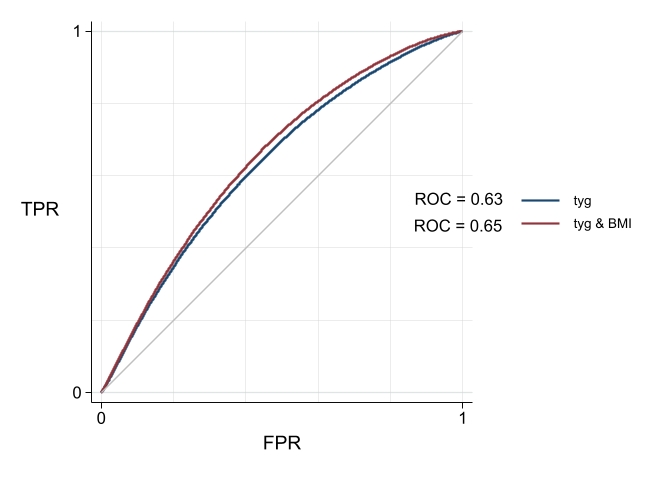


| **Supplementary table 3.** Sensitivity analysis for unmeasured confounding using E-value for odds ratio | | | | |  |
| --- | --- | --- | --- | --- | --- |
|  |  |  |  |  |  |
| **Variables** | **Logistic multivariable analysis** | | **E-value for odds ratio** | |  |
|  | **Adjusted odds ratio** | **95% CI** | **Point estimate** | **Confidence limits** |  |
| **Overall^a^** | | | | |  |
| **TyG index** | 1.55 | 1.52-1.57 | 2.47 | 2.41 |  |
| **TyG index (Quartiles)** |  |  |  |  |  |
| Quartile 1 (<8.37) | ref | ref | ref | ref |  |
| Quartile 2 (8.37-8.78) | 1.45 | 1.41-1.50 | 2.26 | 2.17 |  |
| Quartile 3 (8.79-9.23) | 1.91 | 1.86-1.97 | 3.23 | 3.13 |  |
| Quartile 4 (>9.23) | 2.45 | 2.38-2.52 | 4.31 | 4.19 |  |
| **Male^b^** | | | | |  |
| **TyG index** | 1.53 | 1.50-1.55 | 2.43 | 2.37 |  |
| **TyG index (Quartiles)** |  |  |  |  |  |
| Quartile 1 (<8.37) | ref | ref | ref | ref |  |
| Quartile 2 (8.37-8.78) | 1.41 | 1.37-1.45 | 2.17 | 2.08 |  |
| Quartile 3 (8.79-9.23) | 1.84 | 1.78-1.89 | 3.08 | 2.96 |  |
| Quartile 4 (>9.23) | 2.39 | 2.31-2.46 | 4.21 | 4.05 |  |
| **Female^b^** | | | | |  |
| **TyG index** | 1.94 | 1.80-2.09 | 3.29 | 3.00 |  |
| **TyG index (Quartiles)** |  |  |  |  |  |
| Quartile 1 (<8.37) | ref | ref | ref | ref |  |
| Quartile 2 (8.37-8.78) | 1.79 | 1.60-2.00 | 2.98 | 2.58 |  |
| Quartile 3 (8.79-9.23) | 2.65 | 2.36-2.97 | 4.74 | 4.15 |  |
| Quartile 4 (>9.23) | 2.86 | 2.49-3.27 | 5.17 | 4.42 |  |
| **Obese^c^** | | | | |  |
| **TyG index** | 1.44 | 1.41-1.47 | 2.24 | 2.17 |  |
| **TyG index (Quartiles)** |  |  |  |  |  |
| Quartile 1 (<8.37) | ref | ref | ref | ref |  |
| Quartile 2 (8.37-8.78) | 1.42 | 1.36-1.48 | 2.19 | 2.06 |  |
| Quartile 3 (8.79-9.23) | 1.82 | 1.75-1.90 | 3.04 | 2.90 |  |
| Quartile 4 (>9.23) | 2.26 | 2.16-2.35 | 3.95 | 3.74 |  |
| **Non-obese^c^** | | | | |  |
| **TyG index** | 1.81 | 1.78-1.85 | 3.02 | 2.96 |  |
| **TyG index (Quartiles)** |  |  |  |  |  |
| Quartile 1 (<8.37) | ref | ref | ref | ref |  |
| Quartile 2 (8.37-8.78) | 1.55 | 1.49-1.61 | 2.47 | 2.34 |  |
| Quartile 3 (8.79-9.23) | 2.17 | 2.08-2.26 | 3.76 | 3.58 |  |
| Quartile 4 (>9.23) | 3.06 | 2.93-3.19 | 5.57 | 5.31 |  |
| ^a^Adjusted for age, sex, body mass index, region, scheme, year, smoking status, alcohol drinking, exercise, systolic blood pressure, diastolic blood pressure, aspartate aminotransferase, alanine aminotransferase | | | | |  |
| ^b^Adjusted for age, body mass index, region, scheme, year, smoking status, alcohol drinking, exercise, systolic blood pressure, diastolic blood pressure, aspartate aminotransferase, alanine aminotransferase | | | | |  |
| ^c^Adjusted for age, sex, region, scheme, year, smoking status, alcohol drinking, exercise, systolic blood pressure, diastolic blood pressure, aspartate aminotransferase, alanine aminotransferase | | | | |  |

**Example of E-value interpretation:**

An association between the TyG index and hyperuricemia is observed, with an adjusted odds ratio of 1.55 (1.52-1.57).

The E-value for this point estimate is 2.47.

This E-value suggests that “The observed odds ratio of 1.55 could be attributed to an unmeasured confounder associated with both the TyG index and hyperuricemia, with an odds ratio of at least 2.47-fold, above and beyond the measured confounders. Confounding of a weaker magnitude would not suffice to explain this association” [1].

The E-value for the lower confidence limit is 2.41.

This indicates that 'An unmeasured confounder associated with both the TyG index and hyperuricemia, with an odds ratio of at least 2.41-fold, could account for the lower confidence limit. Weaker confounding would not be sufficient for this explanation” [1].

1. Linden A, Mathur MB, VanderWeele TJ. Conducting sensitivity analysis for unmeasured confounding in observational studies using E-values: The evalue package. The Stata Journal: Promoting communications on statistics and Stata. 2020;20:162–75.
